# Effects of the short-stitch technique for midline abdominal closure: short-term results from the randomised-controlled ESTOIH trial

**DOI:** 10.1007/s10029-021-02410-y

**Published:** 2021-05-28

**Authors:** M. Albertsmeier, A. Hofmann, P. Baumann, S. Riedl, C. Reisensohn, J. L. Kewer, J. Hoelderle, A. Shamiyeh, B. Klugsberger, T. D. Maier, G. Schumacher, F. Köckerling, U. Pession, M. Weniger, R. H. Fortelny

**Affiliations:** 1 Department of General, Visceral and Transplantation Surgery, LMU University Hospital, Ludwig-Maximilians-Universität (LMU) Munich, 81377 Munich, Germany; 2 Allgemein-, Viszeral- und Tumorchirurgie, Wilhelminenspital, Montleartstr. 37, 1160 Vienna, Austria; 3 Department of Medical Scientific Affairs, Aesculap AG, Am Aesculap Platz, 78532 Tuttlingen, Germany; 4 Klinik am Eichert, Allgemeinchirurgie, Alb Fils Klinik GmbH, Eichertstr.3, 73035 Göppingen, Germany; 5 Klinik für Allgemein-, Viszeral- und Gefäßchirurgie, Klinikum Landkreis Tuttlingen, Zeppelinstr. 21, 78532 Tuttlingen, Germany; 6 Klinik für Allgemein- und Viszeralchirurgie, Kepler Universitätsklinikum GmbH, Krankenhausstr. 9, 4021 Linz, Austria; 7 Allgemein- und Viszeralchirurgie, Robert-Bosch-Krankenhaus, Auerbachstr. 110, 70376 Stuttgart, Germany; 8 Chirurgische Klinik, Städtisches Klinikum Braunschweig, Salzdahlumer Str. 90, 38126 Brunswick, Germany; 9 Klinik für Chirurgie, Viszeral- und Gefäßchirurgie, Vivantes Klinikum Spandau, Neue Bergstr. 6, 13585 Berlin, Germany; 10 Zentrum der Chirurgie, Klinik für Allgemein- und Viszeralchirurgie, Universitätsklinikum Frankfurt, Theodor-Stern-Kai, 60590 Frankfurt am Main, Germany; 11 Med. Fakultät, Sigmund Freud Privatuniversität, Freudplatz 3, 1020 Vienna, Austria

**Keywords:** Randomised-controlled trial, Human, Burst abdomen, Surgical site infection, Small bites, Short stitches

## Abstract

**Purpose:**

The short-stitch technique for midline laparotomy closure has been shown to reduce hernia rates, but long stitches remain the standard of care and the effect of the short-stitch technique on short-term results is not well known. The aim of this study was to compare the two techniques, using an ultra-long-term absorbable elastic suture material.

**Methods:**

Following elective midline laparotomy, 425 patients in 9 centres were randomised to receive wound closure using the short-stitch (USP 2-0 single thread, *n* = 215) or long-stitch (USP 1 double loop, *n* = 210) technique with a poly-4-hydroxybutyrate-based suture material (Monomax^®^). Here, we report short-term surgical outcomes.

**Results:**

At 30 (+10) days postoperatively, 3 (1.40%) of 215 patients in the short-stitch group and 10 (4.76%) of 210 patients in the long-stitch group had developed burst abdomen [OR 0.2830 (0.0768–1.0433), *p* = 0.0513]. Ruptured suture, seroma and hematoma and other wound healing disorders occurred in small numbers without differences between groups. In a planned Cox proportional hazard model for burst abdomen, the short-stitch group had a significantly lower risk [HR 0.1783 (0.0379–0.6617), *p* = 0.0115].

**Conclusions:**

Although this trial revealed no significant difference in short-term results between the short-stitch and long-stitch techniques for closure of midline laparotomy, a trend towards a lower rate of burst abdomen in the short-stitch group suggests a possible advantage of the short-stitch technique.

**Trial registry:**

NCT01965249, registered October 18, 2013.

## Introduction

Incisional hernia, which develops in 10–69% of patients, remains a major complication after midline abdominal wall closure [1]. A low tension of the suture line, sufficient collagen deposition, adequate blood supply and the absence of infection are prerequisites for undisturbed wound healing, while clinical risk factors for the development of incisional hernia include obesity, smoking, steroid therapy and malnutrition [1].

The first randomised-controlled trial (RCT) comparing large bite and small bite closure techniques published by Millbourn et al. [2] restarted the discussion regarding the best technique for midline closure. Albeit the INLINE meta-analysis by Diener et al. concluded that a running suture using a long-term absorbable monofilament material should be used for midline closure [3], specific technical aspects were not highlighted. In several studies, Israelsson had investigated the effects of different suture-to-wound length ratios and detected that a ratio of at least 4–5:1 was associated with fewer wound infections and lower incisional hernia rates even in obese patients [4, 5].

In 2015, the European hernia society (EHS) published guidelines on the closure of abdominal wall incisions [6], which included weak recommendations for the use of small bites, based only on the RCT by Millbourn et al. [2], and for a suture-to-wound length ratio of at least 4:0. A second RCT comparing small versus large bite was published by Deerenberg et al. [7] immediately after these guidelines. The results of this study confirmed the superiority of the small bite technique. The surgical site infection (SSI) rate of the STITCH trial was surprisingly high in both groups (21 vs 22%) in comparison to Millbourn (10 vs 5%).

Based on a recently published meta-analysis by Henriksen et al. [8] including 2 RCTs [2, 7], a continuous suture with small bites in combination with a slowly absorbable suture material results in significantly fewer incisional hernias than a large bites technique (9.45 vs 19.30%, OR 0.41, 95% CI 0.19–0.86). These improvements notwithstanding, the surgical site infection rate of 21% and the 1-year incisional hernia rate of 13% in the small bite group of the STITCH study are not satisfactory. Besides stitch length, properties of the suture material such as elasticity, tensile strength and resorption time could significantly influence the results of elective midline closure.

On this account, a multi-centre, international, double-blinded, randomised trial was started to analyse the influence of stitch length on hernia development following elective midline laparotomy closure (ESTOIH study, NCT01965249) [9] using an elastic, extra-long-term absorbable monofilament suture material. Here, we report the short-term results of this trial with a focus on burst abdomen and SSI.

## Methods

### Trial design

This was a multi-centre, double-blinded, controlled, parallel-group study with 1:1 randomisation conducted in Germany and Austria (nine sites). The protocol of this trial has been published previously [9]. After initiation of the trial, changes have been made to the original trial protocol: originally, a body mass index (BMI) ≥ 30 kg/m^2^ had been defined as an exclusion criterion with the intention to keep the study cohort as homogenous as possible. After initiation of the trial, this was found to limit recruitment to the trial. Moreover, Höer et al. had demonstrated that the risk for incisional hernia significantly increases with a BMI > 25 kg/m^2^ [1]. No further difference was seen when the cut-off for BMI was set at 30 kg/m^2^, as most of the high-risk patients are found in the group with BMI < 30 kg/m^2^. Therefore, BMI was dropped from the list of exclusion criteria in an amendment to the study protocol dated 2015-09-23.

Furthermore, the original trial protocol had “pancreatic tumour patients” excluded from the study due to their relatively unfavourable prognosis and the intended 3-year follow-up. In the same amendment, this was changed to “patients undergoing surgery due to a pancreas carcinoma” to allow patients with benign pancreatic tumours to be included in the study.

### Participants

Eligible participants were all adults aged ≥ 18 years (American Society of Anaesthesiologists groups I–III) undergoing an elective, primary median laparotomy with an incision length of ≥ 15 cm and an expected survival time longer than 1 year for whom written consent could be obtained.

Exclusion criteria were emergency surgery, BMI ≥ 30 kg/m^2^, pancreatic tumour patients (cf. “Trial Design” for changes to these exclusion criteria), patients operated due to an abdominal aortic aneurysm, peritonitis, coagulopathy, immunosuppressive therapy at the time of surgery (more than 40 mg of a corticoid per day or azathioprine), chemotherapy within the last 2 weeks before operation, radiotherapy of the abdomen within the last 8 weeks before operation, pregnancy, severe neurologic and psychiatric disease and lack of compliance.

Patients were recruited at nine different trials sites in Germany (seven sites) and Austria (two sites) including three university hospitals, three other tertial referral centres and three local and regional hospitals. The trial started with six centres and three centres joined the group after initiation of the trial.

### Interventions

The main surgical procedures were carried out according to local standards. The linea alba was prepared to be free from subcutaneous fat and cut in the middle. In both study groups, elastic, extra-long-term absorbable, monofilament sutures manufactured from poly-4-hydroxybutyrate (P4HB) (Monomax^®^, B.Braun Surgical, S.A., Rubi, Spain) were used for abdominal wall closure.

In the long-stitch group, a continuous suture with 10 mm stitch intervals and 10 mm distance from the wound edge was performed using a Monomax^®^ USP 1 150 cm loop with an HR 48 mm needle (suture-to-wound length ratio approx. 4:1). Sutures overlapped in the middle and were knotted separately. In the short stich group, a single continuous suture with 5 mm stitch intervals and 5–8 mm distance from the wound edge was performed using a Monomax^®^ USP 2/0 single 150 cm thread with an HR 26 mm needle (suture-to-wound length ratio ≥ 5:1). The number of throws per knot was not standardised in the study protocol. In training sessions, at least six throws for the long-stitch technique and a self-fixing knot for the short-stitch technique were recommended.

Surgeon training included study site visits by the principal investigator (R.F.) and training videos. Data related to suture technique were recorded in the case report form, monitored in regular study site visits and trends were discussed in study group meetings.

### Outcome measures

This analysis reports short-term results of the ESTOIH trial as defined in the published study protocol [9]. The main outcome is the frequency of burst abdomen, defined as a clinically evident rupture of the laparotomy wound, at 30 (+10) days postoperatively. Other short-term results include surgical site infections (SSI), the re-operation rate due to burst abdomen, wound healing disorders, seroma and hematoma within 30 (+10) days. Wound infections are classified according to the US centres for disease control and prevention (CDC) as either deep or superficial. We also analysed the length of hospital stay and complications not directly related to wound healing.

The primary endpoint of the ESTOIH trial is the frequency of incisional hernia 1 year postoperatively and will be reported when available.

### Sample size calculation and interim analysis

The previously published ISSAAC study had found a 19% risk of developing an incisional hernia within 1 year using a long-stitch technique with P4HB-based suture material for abdominal wall closure following primary elective midline laparotomy [10]. The aim of the ESTOIH trial is to demonstrate that the short-stitch suture technique decreases the 1-year incisional hernia rate by 50% compared with the long-stitch technique (primary endpoint). Assuming hernia rates of 19% and 9.5% for the respective groups, a sample size of 424 patients (212 per group) was calculated to detect this difference with a power of 80% and an alpha error of 5%. Including a drop-out rate of 10%, we planned to randomise a total of 468 patients. To avoid centre effects, we determined that no more than 200 patients should be recruited per centre. Withdrawn patients were not to be replaced.

Following an interim analysis of the primary outcome, it was decided that recruitment should end when 424 patients had been randomised as planned in the sample size calculation without substituting for patients who had terminated early.

### Randomisation

Randomisation was performed intraoperatively briefly before abdominal wall closure. Eligible patients were randomly allocated to receive either the short or the long-stitch suture technique in a 1:1 ratio by opening a sealed opaque randomisation envelope. Envelopes were supplied by the sponsor, according to a randomisation list provided by a statistician using the statistical software SAS 9.1 (SAS Institute Inc., Cary, NC, USA). A separate randomisation list was prepared for each participating trial site to avoid centre-specific effects and to assure a balanced distribution of treatments within centres (stratification). Random blocks of different lengths were used. Randomisation lists were sealed and locked up at the sponsor site.

Randomisation envelopes were assigned to patients in chronological order by a surgeon, according to a consecutive and unique randomisation number. Each envelope contained the suture material pertinent to the indented suture technique as well as a description of the technique to be employed for abdominal wall closure. The study site confirmed the randomisation result by sending a fax to the sponsor.

### Blinding

Outcome assessment was double blinded: the patient and the observer responsible for the evaluation of the clinical outcome were unaware of the stitch length used for closing the midline and the observer had no access to the randomisation list. To document the clinical outcome at each follow-up examination, case report forms were handed to the observer by an independent person (e.g. a study nurse). While surgeons performing the abdominal wall closures could not be blinded, they were not involved in outcome assessment.

### Statistical analysis

For this report, data available at the 30 (+10) day follow-up visit were analysed. A planned analysis will be performed after all patients have completed their 1-year follow-up (primary endpoint). Additional analyses will be conducted after completion of the 3- and 5-year follow-up visits. All statistical analyses were done using SAS software version 9.4 (SAS Institute Inc., Cary, NC, USA).

The patient cohort is described as a whole and separately for each treatment group with respect to demographic data and the baseline values of investigated parameters. The secondary endpoints reported here are tabulated as frequencies and rates. Confidence intervals are used when appropriate. The two-sided chi-square test for independent proportions is used to test for independence.

To control for BMI following a protocol amendment that allowed patients with a BMI > 30 kg/m^2^ to participate in this study, Cox proportional hazards models were calculated for burst abdomen and wound infection. A stepped backward elimination method was used for model reduction. The stitch group, BMI and factors with *p* < 0.1 were included in the final model. Statistical significance was defined as a *p* value < 0.05 for all analyses.

## Results

### Patients

Between March 2014 and December 2019, eligible participants were recruited and randomised to receive either the short-stitch (*n* = 215) or long-stitch technique (*n* = 210). The trial ended when 425 patients had been randomised; one additional patient had been included due to simultaneous inclusions in this multi-centre trial.

**Fig. 1:**
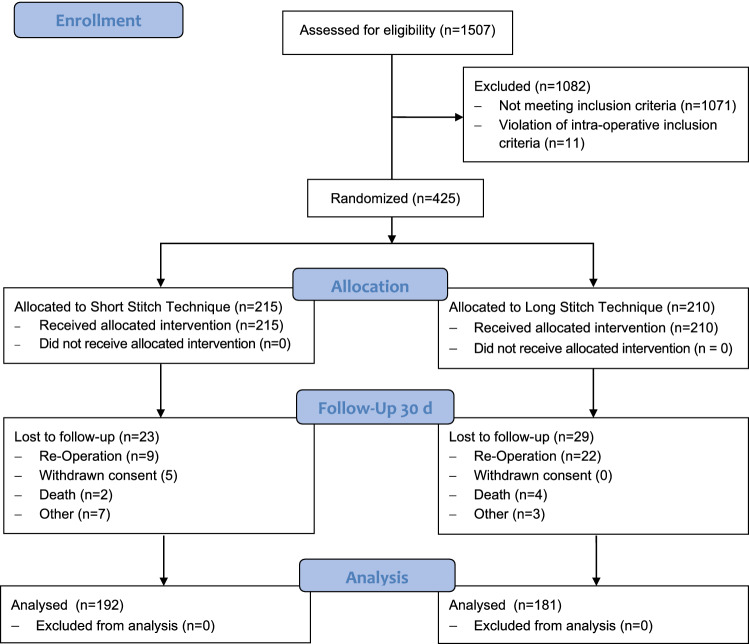
CONSORT flow diagram

The flow of participants through the study is detailed in Fig. 1. Participants received clinical visits at the time of enrolment (baseline), 2 days postoperatively, on the day of discharge and at 30 days (±10 days) postoperatively. Follow-up for short-term results ended January 2020 but continues for incisional hernia. Three hundred seventy-three patients (88%) completed short-term follow-up and were included in the present outcome analysis.

In the short-stitch group, 23 patients were lost to follow-up until day 30 (+10) due to re-operation (*n* = 9), withdrawal of consent (*n* = 5), death (*n* = 2) and other reasons (*n* = 7). In the long-stitch group, 29 patients were lost to follow-up until day 30 (+10) due to re-operation (*n* = 22), death (*n* = 4) and other reasons (*n* = 3).

Study groups were well balanced with respect to baseline clinical and procedure characteristics (Table 1). More than half of patients had colorectal surgery, followed by upper GI surgery. Procedures were performed by 106 different surgeons.

**Table 1:** Baseline demographic and clinical characteristics

	Short stitches *n* (%)	Long stitches *n* (%)
Total	215 (100)	210 (100)
Female gender	100 (47)	93 (44)
BMI (kg/m^2^)	25.4 ± 4.2	25.1 ± 4.1
> 30 kg/m^2^	23 (11)	25 (12)
Smoking		
Previous smoker	33 (15)	36 (17)
Current smoker	36 (17)	32 (15)
Alcohol consumption	44 (21)	64 (30)
ASA classification		
I	31 (14)	38 (16)
II	106 (49)	97 (48)
III	77 (36)	74 (36)
Missing	1 (5)	1 (5)
Type of surgery		
Colon	92 (43)	73 (35)
Oesophagus	8 (4)	7 (3)
Gastric	38 (18)	37 (18)
Pancreas	15 (7)	20 (10)
Rectum	34 (16)	43 (20)
Small intestine	11 (5)	14 (7)
Other	13 (6)	11 (5)
Comorbidities		
Abdominal aortic aneurysm	2 (1)	1 (5)
Chronic liver disease	6 (3)	3 (1)
Diabetes	22 (10)	19 (19)
COPD	12 (6)	8 (4)
Renal insufficiency	12 (6)	7 (3)
Tumour	158 (73)	158 (75)

Data are *n* (%) or mean ± standard deviation

### Outcomes

At 30 (+10) days postoperatively, 3 (1.40%) of 215 patients in the short-stitch group and 10 (4.76%) of 210 patients in the long-stitch group had developed burst abdomen [OR 0.2830 (0.0768–1.0433), *p* = 0.0513]. Ruptured sutures occurred in small numbers of patients with no statistically significant differences between groups. Untied knots were not observed. We found superficial incisional SSIs in seven patients (3.26%) from the short-stitch group and 11 patients (5.24%) from the long-stitch group [OR 0.6088 (0.2314–1.6018), *p* = 0.3440]. Deep incisional SSIs occurred in one patient of each treatment group [0.9766 (0.0607–15.7165), *p* = 1.0000]. Seroma, hematoma and other wound healing disorders were found in small numbers of patients, and no statistically significant differences between groups were observed.

Anastomotic leakage was less frequent in the short-stitch group (6 of 215, 2.79%) compared to the long-stitch group (15 of 210, 7.14%); [OR 0.3787 (0.1441–0.9958), *p* = 0.0460]. 1 (0.47%) of 215 participants in the short-stitch group and 4 (1.90%) of 210 participants in the long-stitch group died in the perioperative period [OR 0.2407 (0.0267–2.1711), n.s.]. Patients in the short-stitch group had shorter hospital stays (11.0 ± 5.0 days) compared to patients in the long-stitch group (12.6 ± 7.8 days, *p* = 0.0204). Outcomes are summarised in Table 2.

**Table 2:** Outcomes: adverse events and length of hospital stay

	Short stitches	Long stitches		
	(*n* = 215)	(*n* = 210)		
	*n* (%)	*n* (%)	Odds ratio	*p*
Burst abdomen	3 (1.40)	10 (4.76)	0.2830 (0.0768–1.0433)	0.0513
SSI				
Superficial	7 (3.26)	11 (5.24)	0.6088 (0.2314–1.6018)	0.344
Deep	1 (0.47)	1 (0.48)	0.9766 (0.0607–15.7165)	1.0000
Hematoma	2 (0.93)	4 (1.90)	0.4836 (0.0876–2.6687)	0.4449
Seroma	6 (2.79)	2 (0.95)	2.9856 (0.5957–14.9632)	0.2847
Wound healing disorder	12 (5.58)	5 (2.38)	2.4236 (0.8387–7.0037)	0.1359
Anastomotic leak	6 (2.79)	15 (7.14)	0.3732 (0.1420–0.9812)	**0.0447**
Ruptured suture	4 (1.86)	2 (0.95)	1.9716 (0.3572–10.8805)	0.6854
Ileus	2 (0.93)	3 (1.43)	0.6479 (0.1072–3.9171)	0.6824
Other	9 (4.19)	11 (5.24)	0.7904 (0.3206–1.9483)	0.6527
Death	1 (0.47)	4 (1.90)	0.2407 (0.0267–2.1711)	0.2111

	Mean (SD)	Mean (SD)	Mean difference	*p*
Hospital stay (days)	11.1 (5.0)	12.6 (7.8)	1.54 (0.24–2.85)	**0.0204**

Bold value indicates *p* value < 0.05 significant

*SSI* surgical site infection

### Cox proportional hazard models

Cox proportional hazards models were calculated for burst abdomen und wound infection (Table 3). In the model for burst abdomen, the short-stitch group had a significantly lower risk compared to the long-stitch group [HR 0.1783 (0.0427–0.7435), *p* = 0.0179]. We observed a trend for a lower risk of burst abdomen with longer fascial closure times. BMI did not increase the risk of burst abdomen. When BMI was removed from the model, the risk of burst abdomen was reduced by 9.7% for every minute that closure of the fascia took longer [HR 1.0965 (1.0094–1.1910), *p* = 0.0291].

**Table 3:** Cox proportional hazard models for burst abdomen and wound infection

Burst abdomen	Hazard ratio	*p*
Short-stitch group	0.1783 (0.0427–0.7435)	**0.0179**
Duration of fascial closure (min)	1.0821 (0.9933–1.1787)	0.0708
BMI > 30 kg/m^2^	2.7147 (0.8092–9.1065)	0.1058

Wound infection	Hazard ratio	*p*
BMI > 30 kg/m^2^	3.0557 (1.0991–8.4947)	**0.0323**
Male gender	3.2958 (1.0926–9.9418)	**0.0342**
Short-stitch group	0.7227 (0.2905–1.7979)	0.4850

Bold value indicates *p* value < 0.05 significant

In the model for wound infection, both BMI > 30 kg/m^2^ [HR 3.0557 (1.0991–8.4947), *p* = 0.0323] and male gender [HR 3.2958 (1.0926–9.9418), *p* = 0.0342] were independently associated with a threefold increased risk of developing the complication. Suture technique had no significant influence on wound infections.

## Discussion

In this analysis of short-term results, we show that closing elective midline laparotomies using a short-stitch technique and an elastic suture material is a safe procedure with a low rate of short-term complications. The rate of burst abdomen did not differ significantly between treatment groups in the primary outcome analysis. In multivariate analysis, however, short stitches were associated with a sevenfold decreased risk for developing burst abdomen.

This is the first study showing such a clear trend towards a reduced risk for burst abdomen; previous trials [2, 7] had shown no difference between suture techniques. The lack of more unanimous conclusions stems from the fact that the ESTOIH trial was not powered for the analysis of short-term wound complications. The overall rate of burst abdomen (3.1%) lies around the upper limit of the range anticipated from previous studies (e.g. PRIMA: 3.3%, INSECT 2.9%, PROUD: 2.6%, STITCH: 1.1%) [7, 11–13], probably reflecting the inclusion of many high-risk oncological surgeries in the present trial (Table 1). In theory, suture material could be an alternative explanation, but this seems unlikely as there were more burst abdomen with the stronger suture. Other outcomes related to wound healing, especially SSIs did not differ between treatment groups. It appears, hence, that if stitch technique did influence healing of the fascia, it did so directly and not primarily via a reduction of wound infections.

Nonetheless, with 4.7% of patients across treatment groups developing an SSI (superficial and deep combined), the rate of wound infections in this trial was low compared to previous trials. In the STITCH trial, e.g. 76 of 560 participants (14.5%) developed superficial or deep SSI [7]. The reason for the low wound infection rate remains unclear: In the ISSAAC trial, which compared P4HB-based sutures (Monomax^®^) to polydioxanone sutures (Monoplus^®^ and PDS^®^), no significant difference in the rate of wound infections was found between suture materials [10]. However, the low rate of wound infections in both treatment groups of the present trial aligns well with results from the MULTIMAC observational cohort study which used P4HB-based suture material in 200 routine patients [14].

The evidence for the use of a monofilament late absorbable running suture is regarded as robust [3], as stated in the EHS guidelines [6] and confirmed in a 2017 Cochrane review [15]. Nonetheless, the physical properties of P4HB-based and polydioxanone-based monofilament threads—i.e. elasticity, basic strength retention and absorption time—differ substantially: the elasticity (elongation) of P4HB-based suture material has been measured to be 90% compared to 45–50% for polydioxanone-based sutures. Presuming that the fascia, not the suture, constitutes the weakest element of abdominal wall closure, increased elasticity might help to reduce the occurrence of button-hole hernia [16–18] at the wound edges. Furthermore, the degradation time (50% basic strength retention) of P4HB-based suture material is 100 days vs 42 and 35 days for polydioxanone-based sutures, respectively, while the mass absorption time of Monomax^®^ is 390 days vs 180–210 days for PDS^®^ and Monoplus^®^. The controlled prospective multi-centre ISSAAC trial [10] showed a non-significant reduction in the combined primary endpoint wound infections and/or burst abdomen in the Monomax^®^ group compared to polydioxanone-based sutures (7.3 vs 11.3%). The authors concluded that Monomax^®^ suture material is as safe as PDS^®^ or Monoplus^®^ for abdominal wall closure after primary midline laparotomy. In summary, P4HB-based suture material seems to support the healing of the fascia by its high elasticity, high basic strength retention and long-lasting resorption time.

In this context, the use of a triclosan-coated slowly absorbable polydioxanone-based suture material has not been successful in decreasing the risk of SSI. A meta-analysis by Henriksen et al. [8] concluded that only multifilament triclosan-coated Vicryl^®^ sutures substantially decrease the risk of SSI following abdominal fascial closure. In the present trial, BMI > 30 kg/m^2^ and male gender but not suture technique were risk factors for developing SSI. In sum, it appears that the development of burst abdomen depends on surgical technique and possibly suture material while wound infections are more related to patient factors.

The surgical technique for fascial closure in the ESTOIH trial was highly standardised using study site and video trainings. Adherence to the study protocol is demonstrated by small deviations from the mean stitch length in both treatment groups (Table 4). The attempted suture-to-wound length ratios were specified based on previous recommendations [5, 6] and they were well adhered to in this study (Table 4).

**Table 4:** Details of suture technique

	Short stitches (*n*=215)	Long stitches (*n*=210)	*p*
Number of stitches (*n*)	45.6 (12.4)	24.9 (7.0)	< **0.001**
Implanted suture length (cm)	113.5 (48.2)	83.1 (26.1)	< **0.001**
Wound length (cm)	21.6 (4.5)	21.4 (4.0)	0.698
Suture length to wound length ratio	5.3 (2.2)	4.0 (1.3)	< **0.001**
Duration of fascial closure (min)	14.9 (5.9)	9.3 (4.1)	< **0.001**

Bold value indicates *p* value < 0.05 significant

Data are mean (SD)

Furthermore, the definition of inclusion and exclusion criteria assured a homogenous patient cohort. Relaparotomy, obesity, abdominal aortic aneurysm, immunosuppression, peritonitis, and emergency surgery had been identified as relevant risk factors for wound healing in previous studies [3, 19–22] and were, therefore, excluded from the present trial. Following slow recruitment, a protocol amendment was introduced early in the trial allowing obese patients to participate. Participants’ BMI, which is the most prevalent confounder in laparotomy trials, was similar between treatment groups (short stitches: 25.4 kg/m^2^ ± 4.2; long stitches: 25.1 kg/m^2^ ± 4.1) and comparable to the previously published STITCH trial (median 24 kg/m^2^ in both groups) [7]. On the other hand, open gynaecological procedures are associated with a significantly reduced risk of hernia development [23, 24] and were excluded for that reason. Together, these strategies contribute to a high internal validity of results from this study.

External validity of the ESTOIH trial was ensured by the multi-centre setting that included community, regional and university hospitals with a large number of participating surgeons. We chose to perform the study in a general surgical population rather than confining it to an extremely high-risk cohort as in the PRIMA trial [22] to maintain generalisability. The inclusion of many colorectal procedures (38.8%) ensured an adequate risk profile of our study cohort [24]. In sum, we believe, that our findings can be generalised to current surgical practice in different situations.

When the short-stitch technique was used, surgeons needed 6 min longer to close abdominal wall, which is acceptable if the procedure proves to be effective. It is an interesting finding of this study, however, that the rate of burst abdomen decreased when the time used for suturing the fascia was longer (independent of stitch technique). This supports the notion that surgical technique is relevant for safe abdominal wall closure.

Admittedly, this trial has some limitations. More patients in the long-stitch group (29 vs 23) dropped out of the study before the visit on day 30, primarily due to re-operations (22 vs 9) which were associated with a greater number of anastomotic leaks (15 vs 6). While it can be excluded that the technique of fascial closure caused these anastomotic leaks or was otherwise the reason for revision surgery except for cases of burst abdomen, the influence of these imbalances on our study results is not clear. The higher drop-out rate in the long-stitch group may have prevented the detection of other complications in these patients.

Furthermore, the longer duration of hospital stays in the long-stitch group may have been caused by the higher frequencies of anastomotic leaks and revision surgery (unrelated to burst abdomen) in this group and not so much by suture technique. Another limitation of this report is the exploratory nature of the secondary outcome analysis for which it was not powered as the predefined primary outcome incisional hernia will be reported when 1-year follow-up has been completed by all patients.

Finally, the long-stitch group was characterised not only by a greater stitch length but also used a double loop suture, which is the current technical standard in many surgical departments. The tissue trauma associated with this technique may be in part a consequence of the double loop with a strong needle and not only stitch length. Using short stitches with a single thread that is a little stronger than USP 2–0 still avoids this kind of tissue trauma and may be a good compromise for those who wish to change their current practice but prefer a more robust suture.

## Conclusion

The short-stitch technique for abdominal wall closure potentially reduces the rate of burst abdomen. Furthermore, ultra-long-term absorbable elastic suture material appears to be associated with low wound infection and overall complication rates. Analysis of long-term results of this trial will help clarify the impact of suture technique on hernia development.

## Data Availability

Individual de-identified participant data will be made available beginning 6 months after publication and ending after 5 years. Data will be shared with investigators who provide a methodologically sound proposal to the sponsor. Proposals shall be directed to petra.baumann@aesculap.de. Data requestors will need to sign a data access agreement. Data are available for 5 years at a third party website. The trial protocol has been published with open access in the journal Trials: Fortelny RH, Baumann P, Thasler WE, et al. Effect of suture technique on the occurrence of incisional hernia after elective midline abdominal wall closure: study protocol for a randomised-controlled trial. Trials 2015; 16(1):52.
